# Comparison of preoxygenation using a tight facemask, humidified high-flow nasal oxygen and a standard nasal cannula – a volunteer, randomised, crossover study

**DOI:** 10.1097/EJA.0000000000001989

**Published:** 2024-04-16

**Authors:** Albin Sjöblom, Magnus Hedberg, Ida-Maria Forsberg, Frida Hoffman, Malin Jonsson Fagerlund

**Affiliations:** From the Perioperative Medicine and Intensive Care, Karolinska University Hospital, Stockholm, Sweden (AS, MH, I-MF, FH, MJF), Department of Physiology and Pharmacology, Section for Anesthesiology and Intensive Care Medicine, Karolinska Institutet, Stockholm, Sweden (AS, MH, I-MF, FH, MJF)

## Abstract

**BACKGROUND:**

Preoxygenation before anaesthesia induction is routinely performed via a tight-fitting facemask or humidified high-flow nasal oxygen. We hypothesised that effective preoxygenation, assessed by end-tidal oxygen (EtO_2_) levels, can also be performed via a standard nasal cannula.

**OBJECTIVE:**

This study compared the efficacy of preoxygenation between a traditional facemask, humidified high-flow nasal oxygen and a standard nasal cannula.

**DESIGN:**

A volunteer, randomised, crossover study.

**SETTING:**

Karolinska University Hospital, Stockholm. The study was conducted between 2 May and 31 May 2023.

**PARTICIPANTS:**

Twenty cardiopulmonary healthy volunteers aged 25–65 years with a BMI <30.

**INTERVENTIONS:**

Preoxygenation using a traditional facemask, humidified high-flow nasal oxygen and standard nasal cannula. Volunteers were preoxygenated with all three methods, at various flow rates (10–50 l min^−1^), with open and closed mouths and during vital capacity manoeuvres.

**MAIN OUTCOME MEASURES:**

The study's primary outcome compared the efficacy after 3 min of preoxygenation, assessed by EtO_2_ levels, between the three methods and various flow rates of preoxygenation.

**RESULTS:**

Three methods generated higher EtO_2_ levels than others: (i) facemask preoxygenation using normal breathing, (ii) humidified high-flow nasal oxygen, closed-mouth breathing, at 50 l min^−1^ and (iii) standard nasal cannula, closed-mouth breathing, at 50 l min^−1^, and expressed as means (SD): 90% (3), 90% (6) and 88% (5), respectively. Preoxygenation efficacy was greater via the bi-nasal cannulae using closed vs. open mouth breathing as well as with 3 min of normal breathing *vs.* eight vital capacity breaths. Preoxygenation with a facemask and humidified high-flow nasal oxygen was more comfortable than a standard nasal cannula.

**CONCLUSION:**

The efficacy of preoxygenation using a standard nasal cannula at high flow rates is no different to clinically used methods today. The standard nasal cannula provides less comfort but is highly effective and could be an option when alternative methods are unavailable.

**TRIAL REGISTRATION:**

Clinicaltrials.gov, NCT05839665.

## Graphical abstract

**Figure:**
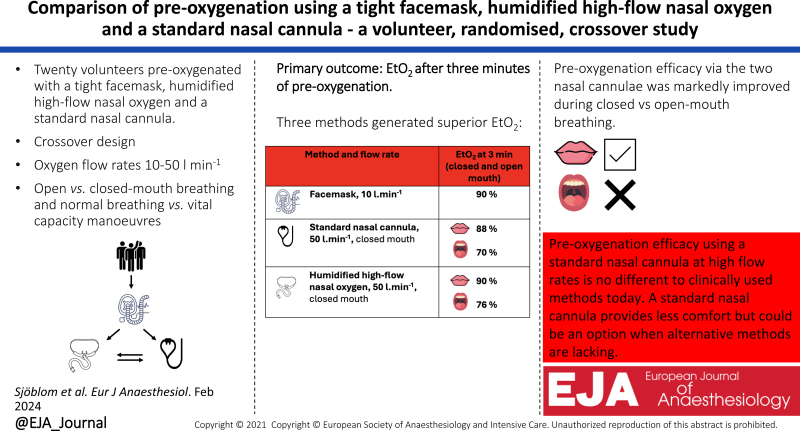

**KEY POINTS**

- A tight-fitting, traditional, facemask and humidified high-flow nasal oxygen are clinically established methods that generate preoxygenation with high efficacy.
- This study, enrolling 20 volunteers, compared preoxygenation efficacy by evaluating end-tidal oxygen levels, between a traditional facemask, humidified high-flow nasal oxygen, and a standard nasal cannula.
- Preoxygenation using a standard nasal cannula, at 50 l min^−1^, generated end-tidal oxygen levels no different to those achieved from normal breathing via a fitting facemask and humidified high-flow nasal oxygen.
- Closed-mouth breathing generated higher preoxygenation efficacy compared to open-mouth breathing when using either of the bi-nasal cannulae.
- Preoxygenation with a facemask and humidified high-flow nasal oxygen was more comfortable compared to a standard nasal cannula.

## Introduction

Preoxygenation is the delivery of oxygen to a spontaneously breathing patient, routinely performed before anaesthesia induction, aiming to replace nitrogen with an oxygen reserve for patients to rely on during apnoea or hypoventilation. Preoxygenation increases the oxygen stored in the functional residual capacity and prolongs the safe apnoea time, which could be crucial during difficult facemask ventilation or tracheal intubation.^1,2^

The efficacy of preoxygenation is typically evaluated by measuring end-tidal oxygen (EtO_2_). Preoxygenation in clinical practice usually consists of breathing 100% oxygen for 3 min via a tight-fitting facemask, aiming to reach EtO_2_ >80% or 90%. Preoxygenation using four to eight vital capacity breaths generates comparable EtO_2_ as 3 min of normal breathing and has been suggested as an alternative method, especially in emergencies, to quickly establish sufficient oxygen reserves.^3–5^

Lately, humidified high-flow nasal oxygen has been investigated and used clinically for preoxygenation with a potential advantage being a seamless transition to apnoeic oxygenation.^6–9^ The effect of preoxygenation using humidified high-flow nasal oxygen is comparable to a tight-fitting facemask,^10^ with data also suggesting potential benefits from using humidified high-flow nasal oxygen regarding anaesthetist-assessed ease and patient comfort.^11^

Oxygen therapy via a standard nasal cannula is inexpensive, available globally, user-friendly and also suitable in the prehospital setting. Flow rates below 10 l min^−1^ are often used due to patient discomfort and mucosal drying. Lately, the standard nasal cannula has been used for apnoeic oxygenation, with flow rates of 5 to 15 l min^−1^, to prolong the safe apnoea time.^12,13^ Recently, it was demonstrated that the standard nasal cannula, at a flow rate of 60 l min^−1^, generated similar pharyngeal expired oxygen concentrations as humidified high-flow nasal oxygen after 3 min of preoxygenation, although with poorer patient comfort.^14^ This study only investigated the standard nasal cannula at a flow rate of 60 l min^−1^. Furthermore, the study could be underpowered since the recruitment of volunteers had to be prematurely terminated and data from only 10 out of the planned 20 participants were analysed.

To our knowledge, no previous study has investigated the standard nasal cannula for preoxygenation at flow rates <60 l min^−1^. The present study was designed to explore the standard nasal cannula and the lower limit of oxygen flow rate that would generate a preoxygenation efficacy comparable to clinically established preoxygenation methods. Furthermore, this study compared open *vs.* closed mouth preoxygenation, 3 min of normal breathing *vs.* vital capacity manoeuvres and investigated time to achieve adequate preoxygenation efficacy.

We hypothesised that effective preoxygenation, assessed by EtO_2_ levels, can also be performed via a standard nasal cannula. Therefore, this study primarily aimed to compare preoxygenation efficacy, assessed by EtO_2_ levels, using a tight-fitting facemask, humidified high-flow nasal oxygen and a standard nasal cannula, at different oxygen flow rates.

## Methods

This study was approved by the Swedish Medical Products Agency (CIV-22-09-040826), Uppsala, Sweden on 9 March 2023 (Chairperson Erika Edman) and the Swedish Ethical Review Authority (Dnr 2022-06747-01), Uppsala, Sweden on 28 February 2023 (Chairperson Mattias Nordell). The study followed the declaration of Helsinki and was registered at clinicaltrials.gov (NCT05839665) before volunteer enrolment. All participants received oral and written information before signing a consent form. An independent study monitor conducted regular audits during the study period.

The study was conducted at the Karolinska University Hospital, Stockholm, between 2 May and 31 May 2023, and recruited volunteers between the ages of 25 and 65 years. Exclusion criteria were BMI >30, cardiac or pulmonary comorbidity, current smoking, and pregnancy.

Volunteers were placed supine and a three-lead ECG and pulse oximetry monitoring (Philips Intellivue MX800, X3) were applied. All volunteers performed 18 cycles of preoxygenation in total, comprising three different apparatus, and varying flow, using both open and closed-mouth breathing and 3 min of normal breathing as well as vital capacity manoeuvres.

**Fig. 1:**
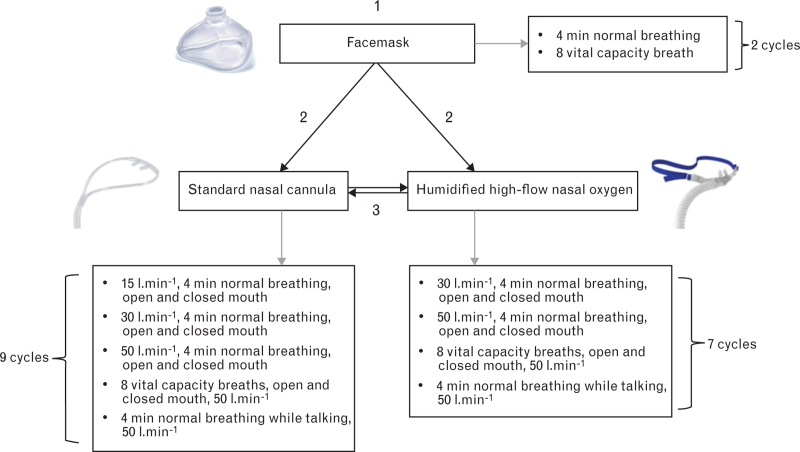
Study protocol.

The study protocol included preoxygenation with three different techniques: tight facemask, humidified high-flow nasal oxygen and standard nasal cannula (Fig. 1). During all cycles and methods, 100% oxygen was delivered. Between every cycle, volunteers were breathing room air for at least 2 min to ensure EtO_2_ <20% before initiating the next cycle. End-tidal oxygen was measured via a sampling line at the expiratory end of a circle system (FLOW-i, Maquet Critical Care AB, Solna, Sweden/Avance CS^2^, GE Healthcare, Waukesha, WI, USA).

All volunteers started with facemask preoxygenation (1) and were thereafter randomised to preoxygenation using humidified high-flow nasal oxygen or the standard nasal cannula (2). Lastly, all volunteers were preoxygenated with the method remaining (3).

All volunteers were first preoxygenated with a tight-fitting facemask connected to a ventilator via a circle system (FLOW-i, Maquet Critical Care AB, Solna, Sweden/Avance CS^2^, GE Healthcare, Waukesha, WI, USA) (Fig. 1). The facemask delivered 100% oxygen and was held by an anaesthetist to ensure a tight seal to avoid leakage. First, volunteers were instructed to breathe normally for 4 min, with the ventilator delivering a fresh gas flow of 10 l min^−1^. Thereafter, a vital capacity manoeuvre consisting of eight vital capacity breaths with the ventilator delivering a fresh gas flow of 20 l min^−1^ was performed (Fig. 1).

Following facemask preoxygenation, volunteers were randomised to preoxygenation using humidified high-flow nasal oxygen (Optiflow TM, Fisher and Paykel Healthcare, Auckland, New Zealand) or the standard nasal cannula (Nasal Cannula Soft, Mediplast AB, Malmö, Sweden). Randomisation was performed by a study group member using sealed envelopes assigned in a 1 : 1 ratio in blocks of four. Lastly, volunteers were preoxygenated with the method remaining after randomisation.

Preoxygenation using humidified high-flow nasal oxygen was performed in cycles of 4 min and flow rates of 30 and 50 l min^−1^, with both open and closed mouth at each flow rate. Thereafter, volunteers performed vital capacity manoeuvres, consisting of eight breaths, at a flow rate of 50 l min^−1^, with both open and closed mouth. Lastly, volunteers performed one cycle of preoxygenation at 50 l min^−1^, while reading a text for 4 min to simulate a talking patient during preoxygenation.

Preoxygenation with the standard oxygen nasal cannula was performed with the same protocol as humidified high-flow nasal oxygen, with the addition of two cycles with a flow rate of 15 l min^−1^, one with an open and one with a closed mouth.

The allocation of preoxygenation within each interface was done sequentially in the same way for each participant (Fig. 1).

During preoxygenation with humidified high-flow nasal oxygen and the standard nasal cannula, EtO_2_ was measured once every minute (1, 2, 3 and 4 min). Volunteers were instructed to hold their breath at the end of a normal inhalation. Oxygen flow was then turned off and volunteers exhaled into the circle system, connected to the ventilator. Following exhalation, volunteers were instructed to hold the exhalation and continue breathing only after oxygen flow was turned on. This procedure was overseen by the study group to ensure it was performed accurately. During vital capacity manoeuvres with the bi-nasal cannulae, EtO_2_ was measured only after the eighth breath.

A gas flow analyser (VT PLUS HF, FLUKE Biomedical, Cleveland, USA) was used to ensure correct oxygen flow from the ventilator, the humidified high-flow nasal oxygen system and the standard nasal cannula.

After each cycle of preoxygenation volunteers were asked to assess the level of discomfort on a scale between 1 to 10 (1 = no discomfort, 5 = moderated discomfort, 10 = severe discomfort) and to report any adverse events.

### Outcomes

The study's primary outcome compared the efficacy after 3 min of preoxygenation, assessed by EtO_2_ levels, between the three methods and various flow rates of preoxygenation.

Secondary outcomes investigated differences in: (i) EtO_2_ when preoxygenation was performed with open or closed mouth, (ii) the duration of preoxygenation to reach EtO_2_ above 80% and 85%, (iii) EtO_2_ between 3 min of normal breathing and vital capacity manoeuvre and (iv) comfort between the three methods and various flow rates.

### Statistics

Based on previous work by us and others, we assumed that a 5% difference in EtO_2_ after 3 min of preoxygenation would be reasonable and clinically relevant. We estimated that the standard deviation in EtO_2_ after 3 min of preoxygenation at most would be 6 units and that the variation for each of the different methods would be similar. Using a type-1 error of 5% and a type-2 error of 20% (power 80%), a sample size of 13 volunteers was required. To adjust for missing data and dropouts, we aimed to include 20 volunteers.

Repeated measures ANOVA were used to assess differences in the primary outcome. When the assumption of sphericity was violated, as assessed by Mauchly's Test of Sphericity, a Greenhouse–Geisser correction was applied. If statistically significant differences were shown, *post hoc* analysis using Fisher's Least Significant Difference (LSD) test was performed. For secondary outcomes, paired or unpaired *t*-tests analysed differences between groups as appropriate. The Friedman test was used to compare the comfort level during preoxygenation. *Post hoc* analysis was done using a Wilcoxon signed-rank test.

SPSS Statistics 28 (IBM, Armonk, NY, USA) was used for statistical tests and Prism 9.0 (GraphPad, Software Inc., San Diego, USA) to create figures.

## Results

Twenty volunteers, eleven males (55%) and nine females (45%), with ASA physical status classification of 1–2 were included in the study (see Figure, Supplemental Digital Content 1, which demonstrates the study flow chart). The mean (SD) age and BMI were 40 (13) years and 23 (3) kg m^−2^. A 2-min washout period sufficiently returned EtO_2_ values to baseline in each volunteer and all preoxygenation cycles (data not shown). This was confirmed before every cycle of preoxygenation. One volunteer was excluded from the primary outcome analysis due to missing data. Data from 19 volunteers were therefore assessed.

### Primary outcome

End-tidal oxygen levels after 3 min of preoxygenation with the different methods and flow rates are presented in Table 1 and Supplemental Digital Content 2. Three methods generated higher EtO_2_ than the rest: (i) facemask preoxygenation using 3 min of normal breathing, (ii) humidified high-flow nasal oxygen, closed-mouth breathing, at 50 l min^−1^ and (iii) standard nasal cannula, closed-mouth breathing, at 50 l min^−1^, 90% (3), 90% (6) and 88% (5), mean (SD), respectively. There was no difference in EtO_2_ levels between these three techniques, facemask vs. humidified high-flow nasal oxygen (*P* = 0.943), facemask vs. standard nasal cannula (*P* = 0.883) or humidified high-flow nasal oxygen vs. the standard nasal cannula (*P* = 0.369).

**Table 1:** End-tidal oxygen after 3 min of preoxygenation or eight vital capacity breaths with the different methods and flow rates and percentage of volunteers with end-tidal oxygen above 80% and 85% after 1, 2 and 3 min or 8 vital capacity breaths of preoxygenation

		Percentage of volunteers with EtO_2_ >80%/85%			
Method and flow rate	EtO_2_ (%) at 3 min Mean ± SD [Min–Max]	1 min	2 min	3 min	8 vital capacity breaths
*Facemask*					
Normal breathing	90 ± 3 [85–94]	15/10	80/55	100/90	
8 vital capacity breaths	90 ± 4 [80–93]	–	–	–	95/90
*Humidified high-flow nasal oxygen*					
30 l min^−1^ open mouth	73 ± 12 [56–90]	10/5	25/15	30/25	–
30 l min^−1^ closed mouth	86 ± 8 [69–94]	40/10	65/55	75/70	–
50 l min^−1^ open mouth	76 ± 16 [43–95]	35/15	40/25	50/30	–
50 l min^−1^ closed mouth	90 ± 6 [72–96]	45/25	100/70	90/85	–
8 vital capacity breaths 50 l min^−1^ open mouth	63 ± 10 [47–80]	–	–	–	0/0
8 vital capacity breaths 50 l min^−1^ closed mouth	83 ± 9 [66–94]	–	–	–	65/45
Talking 50 l min^−1^	67 ± 13 [52–93]	5/0	10/5	15/10	–
*Standard nasal cannula*					
15 l min^−1^ open mouth	61 ± 8 [45–73]	0/0	0/0	0/0	–
15 l min^−1^ closed mouth	73 ± 7 [59–81]	5/0	5/0	20/5	–
30 l min^−1^ open mouth	65 ± 10 [46–81]	0/0	5/0	5/0	–
30 l min^−1^ closed mouth	84 ± 7 [72–94]	20/15	40/25	65/35	–
50 l min^−1^ open mouth	70 ± 13 [35–86]	10/0	5/0	15/5	–
50 l min^−1^ closed mouth	88 ± 5 [81–96]	45/20	90/55	100/65	–
8 vital capacity breaths 50 l min^−1^ open mouth	59 ± 12 [37–77]	–	–	–	0/0
8 vital capacity breaths 50 l min^−1^ closed mouth	78 ± 11 [57–95]	–	–	–	45/30
Talking 50 l min^−1^	62 ± 15 [28–88]	0/0	10/5	10/5	–

### Secondary outcomes

#### Open vs. closed mouth breathing

Closed-mouth breathing was superior to open-mouth breathing (Table 2) (see Figure, Supplemental Digital Content 2, which demonstrates EtO_2_ values during open and closed-mouth breathing). Open-mouth breathing did not generate EtO_2_ >80% at any flow rate with either humidified high-flow nasal oxygen or the standard nasal cannula. Closed-mouth breathing generated EtO_2_ >80% already after 2 min, with both nasal cannulae at flow rates ≥30 l min^−1^. Talking during preoxygenation did not generate EtO_2_ above 70% with either of the bi-nasal cannulae (Table 1); see Figure, Supplemental Digital Content 2, which demonstrates EtO_2_ values when talking during preoxygenation.

**Table 2:** Comparison of end-tidal oxygen levels after 3 min of preoxygenation with open and closed mouth

Method and flow rate	EtO_2_ (%) at 3 min Mean ± SD [Min - Max]	*P*-value
*Humidified high-flow nasal oxygen*		
30 l min^−1^ open mouth	73 ± 12 [56–90]	<0.001
30 l min^−1^ closed mouth	86 ± 8 [69–94]	
50 l min^−1^ open mouth	76 ± 16 [43–95]	0.003
50 l min^−1^ closed mouth	90 ± 6 [72–96]	
*Standard nasal cannula*		
15 l min^−1^ open mouth	61 ± 8 [45–73]	<0.001
15 l min^−1^ closed mouth	73 ± 7 [59–81]	
30 l min^−1^ open mouth	65 ± 10 [46–81]	<0.001
30 l min^−1^ closed mouth	84 ± 7 [72–94]	
50 l min^−1^ open mouth	70 ± 13 [35–86]	<0.001
50 l min^−1^ closed mouth	88 ± 5 [81–96]	

#### Duration of preoxygenation until end-tidal oxygen above 80% and 85%

Most volunteers reached EtO_2_ >85% after 2 min with the methods generating the highest EtO_2_ levels (facemask using normal breathing, humidified high-flow nasal oxygen, closed-mouth breathing at 50 l min^−1^ and standard nasal cannula, closed-mouth breathing at 50 l min^−1^) (Table 1).

End-tidal oxygen increased, for most methods, between 1 vs. 2 min and 2 vs. 3 min of preoxygenation (see Figure, Supplemental Digital Content 2, which demonstrates EtO_2_ over time during preoxygenation) (see Table, Supplemental Digital Content 3, which presents EtO_2_ values at 1, 2, 3 and 4 min of preoxygenation). During facemask preoxygenation using normal breathing and humidified high-flow nasal oxygen, closed-mouth breathing at 30 l min^−1^, EtO_2_ was higher after 4 vs. 3 min expressed as means (SD): 92% (2) vs. 90% (3), *P* < 0.001 and 87% (7) vs. 86% (8), *P* = 0.009), respectively, however for all other methods no further increase in EtO_2_ between 3 and 4 min of preoxygenation was seen (see Table, Supplemental Digital Content 3, which presents EtO_2_ values at 1, 2, 3 and 4 min of preoxygenation).

#### Normal breathing vs. vital capacity manoeuvre

**Fig. 2:**
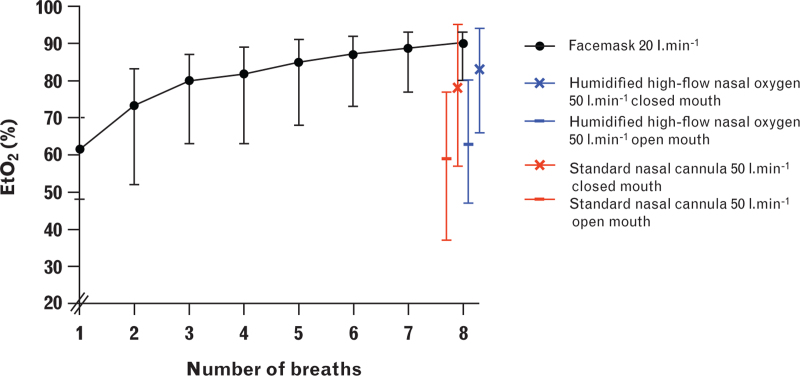
End-tidal oxygen during vital capacity manoeuvres.

Eight vital capacity breaths via the facemask generated a mean EtO_2_ concentration of 90% (SD 4), no different from facemask using 3 min of normal breathing or the bi-nasal cannulae, using closed-mouth breathing at 50 l min^−1^ (Fig. 2) (see Table, Supplemental Digital Content 4, which compares normal breathing to vital capacity breathing). Seven vital capacity breaths achieved EtO_2_ levels comparable to 3 min of normal breathing via the facemask. Mean (SD) end-tidal oxygen was higher after eight vital capacity breaths compared to 2 min of normal breathing: 90% (4) vs. 86% (5) (*P* < 0.001). Eight vital capacity breaths via the facemask had a mean (SD) duration of 72 (23) s. In 11 volunteers, the peak inspiratory flow was measured during facemask preoxygenation using vital capacity manoeuvres. The mean (SD) peak inspiratory flow for these 11 volunteers was 80 (27) l min^−1^.

End-tidal oxygen is plotted for each vital capacity breath for facemask preoxygenation and after eight vital capacity breaths during preoxygenation using humidified high-flow nasal oxygen and standard nasal cannula with both open and closed mouth. Due to technical reasons, EtO_2_ could not be measured at each breath for the nasal cannulae. Graph demonstrates mean and range.

Vital capacity manoeuvres were inferior to normal breathing with humidified high-flow nasal oxygen and the standard nasal cannula, regardless of open or closed-mouth breathing (see Table, Supplemental Digital Content 4).

#### Comfort assessment

**Fig. 3:**
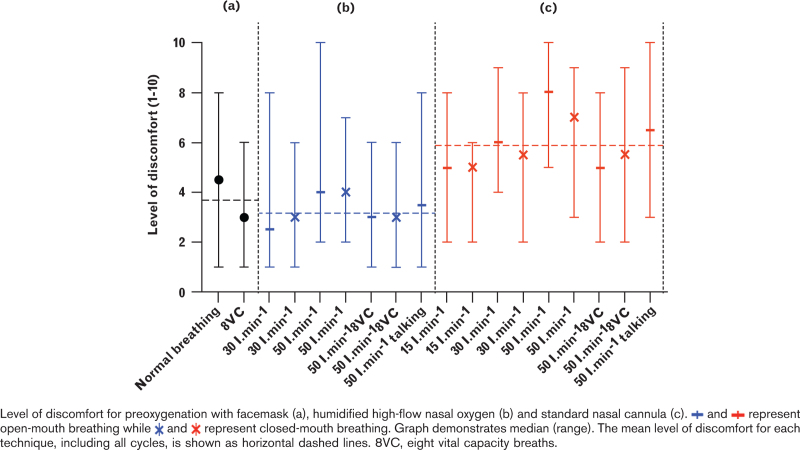
Level of discomfort.

The comfort level was compared between the methods of preoxygenation generating the highest EtO_2_. Expressed as medians (IQR) [range], respectively, the standard nasal cannula at 50 l min^−1^ with a closed mouth was more uncomfortable than both facemask using 3 min of normal breathing; 7 (6–8) [3–9] vs. 5 (2–6) [1–8], *P* = 0.001; and humidified high-flow nasal oxygen at 50 l min^−1^ with a closed mouth 7 (6–8) [3–9] vs. 4 (3–5) [2–7], *P* < 0.001 (Fig. 3). No differences were seen between facemask preoxygenation using 3 min of normal breathing and humidified high-flow nasal oxygen at 50 l min^−1^ (*P* = 0.418). Using the standard nasal cannula, closed-mouth breathing was more comfortable than open-mouth breathing, at all flow rates (Fig. 3). No differences in comfort levels between open and closed-mouth breathing were seen using humidified high-flow nasal oxygen (Fig. 3). Four volunteers reported headaches using the standard nasal cannula at 30 and 50 l min^−1^, which spontaneously resolved once oxygen delivery was terminated.

## Discussion

This randomised, crossover study, showed that preoxygenation using a standard nasal cannula at 50 l min^−1^ generates EtO_2_ comparable to a tight-fitting facemask and humidified high-flow nasal oxygen. Four methods of preoxygenation were superior: facemask using 3 min of normal breathing, facemask using eight vital capacity breaths, humidified high-flow nasal oxygen, breathing with a closed mouth at 50 l min^−1^ and the standard nasal cannula, breathing with a closed mouth at 50 l min^−1^.

This study confirms the results from a recent volunteer study where the efficacy of preoxygenation using a standard nasal cannula at flow rates of 60 l min^−1^ was equal to preoxygenation using humidified high-flow nasal oxygen.^14^ In our study, we compared the most used preoxygenation techniques in clinical practice at different flow rates. Previous data has shown that a tight fitting facemask and humidified high-flow nasal oxygen generate effective preoxygenation.^7,10,15,16^ To our knowledge, no previous study demonstrates that a standard nasal cannula at high flow rates might be an alternative method, equally effective as a facemask and humidified high-flow nasal oxygen. In our study, we show that a flow rate of 50 l min^−1^ using the standard nasal cannula is necessary to achieve a preoxygenation efficacy comparable to a standard facemask and humidified high-flow nasal oxygen. However, when breathing with a closed mouth, the standard nasal cannula generates EtO_2_ levels >80% for most of the volunteers at flow rates at only 30 l min^−1^. Preoxygenation via the standard nasal cannula was more uncomfortable than facemask and humidified high-flow nasal oxygen. Therefore, when available, preoxygenation with a facemask or humidified high-flow nasal oxygen may be preferred in routine clinical practice. However, since the standard nasal cannula is user-friendly for the clinician, easy to set up and transfer, widely available and inexpensive it is of great value when alternative methods are unavailable, especially in clinical emergencies.

This study shows that EtO_2_ >85% can be attained after 2 min with either a facemask using normal breathing or the bi-nasal cannulae using closed-mouth breathing at 50 l min^−1^. By extending the preoxygenation to 3 or 4 min, minor increases in EtO_2_ were observed for some methods, although with questionable clinical significance. To account for individual variation, the often used recommendation of 3 min for preoxygenation therefore seems reasonable and is also valid for preoxygenation with a standard nasal cannula and humidified high-flow nasal oxygen.

This study demonstrates that preoxygenation efficacy with bi-nasal cannulae is markedly improved with closed-mouth compared to open-mouth breathing. Open-mouth breathing did not generate EtO_2_ >80% at any flow rate. These results align with a previous study comparing open- to closed-mouth preoxygenation using humidified high-flow nasal oxygen.^7^ Even though oxygen flow rates greatly exceed peak inspiratory flow during normal breathing, a significant amount of room air is probably entrained during open-mouth breathing. In previous clinical studies investigating preoxygenation with humidified high-flow nasal oxygen, study subjects decided whether to breathe with open or closed mouths which may have affected the results.^8,9,17^ Talking during preoxygenation was one of the least effective methods. This highlights the importance of limiting the number of questions asked to a patient during preoxygenation.

Previous data suggest that four to eight vital capacity breaths via a tight-fitting facemask generate EtO_2_ levels comparable to facemask preoxygenation using 3 min of normal breathing.^3–5^ In this study, seven vital capacity breaths were required to achieve EtO_2_ levels comparable to facemask preoxygenation using 3 min of normal breathing. This aligns with older studies demonstrating a shorter time to desaturation after four vital capacity breaths compared to 3 min of normal breathing.^18,19^ The duration of the vital capacity manoeuvre was less than half the time of 3 min of normal breathing which supports the idea that a vital capacity manoeuvre consisting of eight breaths could be an alternative when time is limited. The efficacy of vital capacity manoeuvres via the bi-nasal cannulae was poor, especially during open-mouth breathing, which may be due to entrained air and reduced FiO_2_ because of a peak inspiratory flow exceeding the oxygen flow rate.

Finally, we assessed the level of discomfort for the different methods. After preoxygenation with a traditional facemask, volunteers were randomised to any of the bi-nasal cannulae to avoid introducing bias and skewness in the comfort assessment by having all volunteers start with the same nasal cannula. Preoxygenation using the standard nasal cannula generated the most discomfort, in line with a study comparing preoxygenation using humidified high-flow nasal oxygen and a standard nasal cannula in healthy volunteers.^14^ Interestingly, closed-mouth preoxygenation was not more uncomfortable than an open mouth. The comfort of the standard nasal cannula was even superior during closed-mouth vs. open-mouth breathing. Therefore, we suggest that preoxygenation, with any of the bi-nasal cannulae, should be performed with a closed mouth since it is highly more effective and more comfortable than open-mouth breathing.

### Limitations

There are some limitations to our study. Volunteers with a BMI above 30 or cardiopulmonary comorbidity were excluded. Since most volunteers were relatively young, with a normal BMI and cardiopulmonary healthy our results may not be extrapolated to other populations. While no volunteer reported prior issues with nasal obstruction, they were not explicitly asked about their nasal patency. Whether minor nasal obstruction in any of the volunteers could affect the preoxygenation effectiveness with any of the bi-nasal cannulae therefore remains uncertain.

All volunteers conducted a total of 18 cycles of preoxygenation. Moreover, all volunteers commenced with facemask preoxygenation, and there was no randomisation performed concerning the sequence of preoxygenation cycles within each of the three methods. Although this was a cross-over study the comfort assessment after each cycle of preoxygenation could be influenced by the total number of preceding cycles and not fully reflect the most recent one.

Comfort level evaluations were only conducted directly following each cycle of preoxygenation, and no long-term assessment of potential adverse effects was performed. The use of high flows of dry oxygen could theoretically result in lasting side effects, such as anosmia and sinusitis. In the context of preoxygenation, we nonetheless believe that the risk of any long term damage to the mucosal tissue is low, considering the brief duration of exposure.

This study used end-tidal oxygen to assess the primary outcome, rather than PaO_2,_ due to the less invasive nature of the measurement.

Considering that this study included a restricted number of volunteers and encompassed several secondary outcomes, these outcomes should be interpreted in light of their exploratory nature. Nevertheless, for the majority of secondary outcomes, there was notable homogeneity in the data. We firmly believe that these findings contribute valuable information and raise questions for future research.

Lastly, the study focused exclusively on preoxygenation; one of two fundamental manoeuvres available to prolong the safe apnoea time. The subsequent manoeuvre, performed when breathing has ceased, is apnoeic oxygenation. This study shows that preoxygenation with a standard nasal cannula at high flow rates is as effective as preoxygenation using a facemask. A follow-up study combining pre and apnoeic oxygenation, using the standard nasal cannula, would be of great value.

## Conclusion

In this volunteer, randomised, crossover study investigating preoxygenation, we could not show any differences in the efficacy of preoxygenation when using a standard nasal cannula with a closed mouth at 50 l min^−1^, a tight-fitting facemask or humidified high-flow nasal oxygen. Even though the standard nasal cannula provides less comfort than a tight-fitting facemask and humidified high-flow nasal oxygen, it is highly effective and could therefore be an option when alternative methods are lacking.

## Supplementary Material

Supplemental Digital Content

## Supplementary Material

Supplemental Digital Content

## Supplementary Material

Supplemental Digital Content

## Supplementary Material

Supplemental Digital Content
