# Supplementary figures and images for: Comparison of preoxygenation using a tight facemask, humidified high-flow nasal oxygen and a standard nasal cannula – a volunteer, randomised, crossover study
Source: Eur J Anaesthesiol. 2024 Apr 16;41(6):430–7. doi: 10.1097/EJA.0000000000001989 (PMC11064899; doi:10.1097/EJA.0000000000001989)

## Slide 1
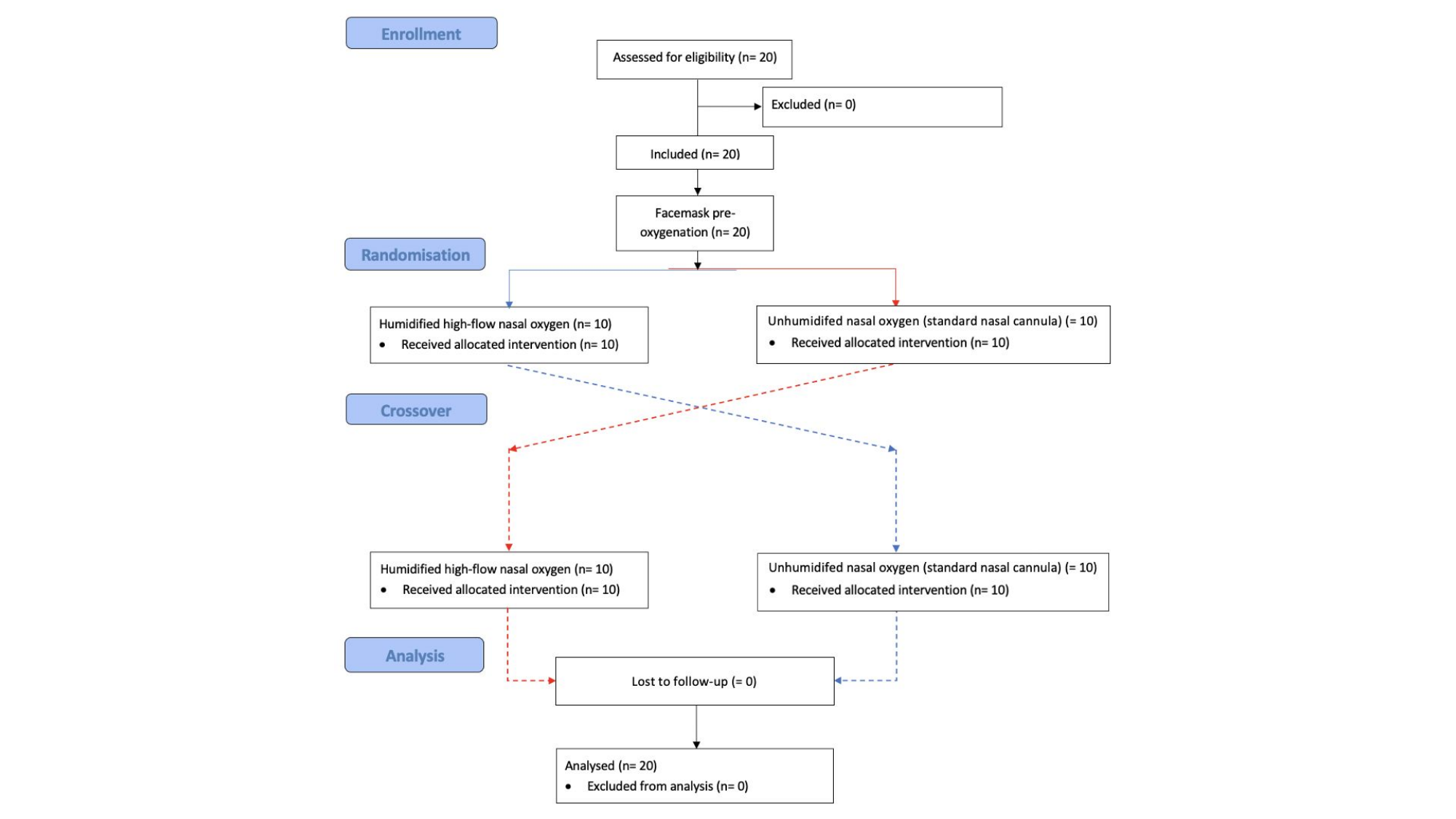

Supplement: Supplemental Digital Content [file ejanet-41-430-s001.pptx]

## Slide 1
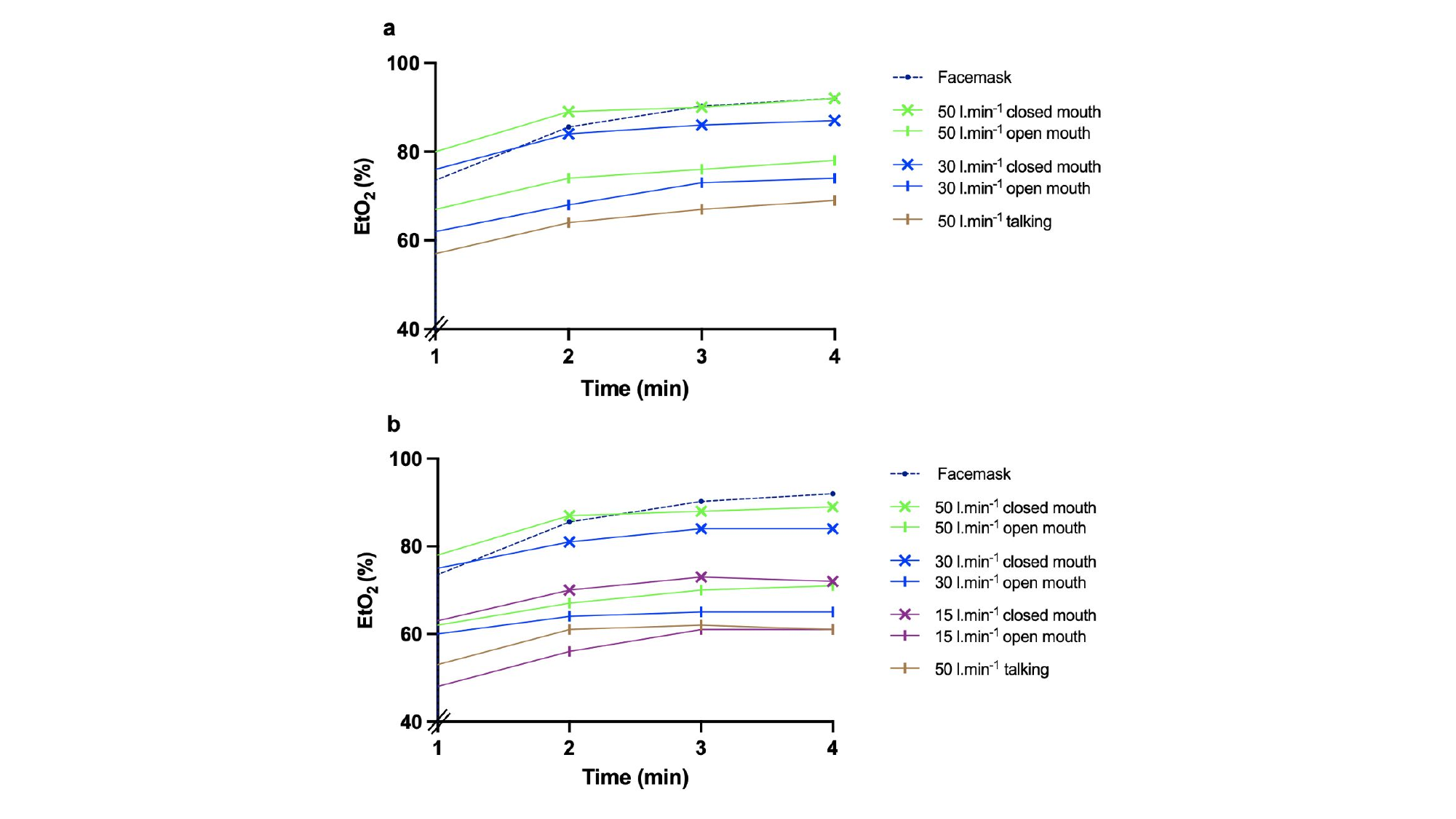

Supplement: Supplemental Digital Content [file ejanet-41-430-s002.pptx]
