# Supplementary material for: Comparison of preoxygenation using a tight facemask, humidified high-flow nasal oxygen and a standard nasal cannula – a volunteer, randomised, crossover study
Source: Eur J Anaesthesiol. 2024 Apr 16;41(6):430–7. doi: 10.1097/EJA.0000000000001989 (PMC11064899; doi:10.1097/EJA.0000000000001989)
Supplement: Supplemental Digital Content [file ejanet-41-430-s003.docx]

**Table, Supplemental Digital Content 3**

End-tidal oxygen after 1, 2, 3 and 4 minutes of pre-oxygenation. *P*-values are derived from a comparison between 1 *vs.* 2 minutes, 2 *vs.* 3 minutes and 3 *vs.* 4 minutes for every method.

| **Method and flow rate** | **EtO_2_ (%) at 3 min**  Mean ± SD | | | | ***P* – value** | | |
| --- | --- | --- | --- | --- | --- | --- | --- |
|  | 1 min | 2 min | 3 min | 4 min | 1 vs 2 min | 2 vs 3 min | 3 vs 4 min |
| *Facemask* |  | | | |  | | |
| Normal breathing | 74 ± 8 | 86 ± 5 | 90 ± 3 | 92 ± 2 | <0.001 | <0.001 | <0.001 |
| *Humidified high-flow nasal oxygen* |  | | | |  | | |
| 30 l min^-1^ open mouth | 62 ± 14 | 68 ± 12 | 73 ± 12 | 74 ± 13 | 0.014 | 0.054 | 0.841 |
| 30 l min^-1^ closed mouth | 76 ± 9 | 84 ± 8 | 86 ± 8 | 87 ± 7 | <0.001 | <0.001 | 0.009 |
| 50 l min^-1^ open mouth | 67 ± 18 | 74 ± 14 | 76 ± 16 | 78 ± 16 | 0.083 | 0.306 | 0.607 |
| 50 l min^-1^ closed mouth | 80 ± 7 | 89 ± 5 | 90 ± 6 | 92 ± 5 | <0.001 | <0.001 | 0.102 |
| 50 l min^-1^ talking | 57 ± 12 | 64 ± 14 | 70 ± 13 | 69 ± 11 | 0.006 | 0.149 | 0.310 |
| *Standard nasal cannula* |  | | | |  | | |
| 15 l min^-1^ open mouth | 48 ± 11 | 56 ± 8 | 61 ± 8 | 61 ± 10 | <0.001 | <0.001 | 0.943 |
| 15 l min^-1^ closed mouth | 63 ± 10 | 71 ± 8 | 73 ± 7 | 72 ± 9 | <0.001 | 0.174 | 0.525 |
| 30 l min^-1^ open mouth | 60 ± 14 | 64 ± 11 | 65 ± 10 | 65 ± 10 | 0.151 | 0.016 | 0.946 |
| 30 l min^-1^ closed mouth | 75 ± 8 | 81 ± 8 | 84 ± 7 | 84 ± 8 | <0.001 | <0.001 | 0.958 |
| 50 l min^-1^ open mouth | 62 ± 16 | 67 ± 13 | 70 ± 13 | 71 ± 10 | 0.083 | 0.100 | 0.361 |
| 50 l min^-1^ closed mouth | 78 ± 8 | 87 ± 6 | 88 ± 5 | 89 ± 5 | <0.001 | <0.001 | 0.254 |
| 50 l min^-1^ talking | 53 ± 14 | 61 ± 15 | 62 ± 15 | 61 ± 16 | 0.028 | 0.350 | 0.709 |
