# Supplementary material for: Comparison of preoxygenation using a tight facemask, humidified high-flow nasal oxygen and a standard nasal cannula – a volunteer, randomised, crossover study
Source: Eur J Anaesthesiol. 2024 Apr 16;41(6):430–7. doi: 10.1097/EJA.0000000000001989 (PMC11064899; doi:10.1097/EJA.0000000000001989)
Supplement: Supplemental Digital Content [file ejanet-41-430-s004.docx]

**Table, Supplemental Digital Content 4**

Comparison of end-tidal oxygen levels using three minutes of normal breathing and eight vital capacity breaths.

| **Method and flow rate** | **EtO_2_ (%) at 3 min**  Mean ± SD [Min - Max] | ***P* - value** |
| --- | --- | --- |
| *Facemask* |  |  |
| Normal breathing | 90 ± 3 [85 - 94] | 0.795 |
| 8 vital capacity breaths | 90 ± 4 [80 - 93] |  |
| *Humidifed high-flow nasal oxygen* |  |  |
| 50 l min^-1^ open mouth | 76 ± 16 [43 - 95] | <0.001 |
| 8 vital capacity breaths  50 l min^-1^ open mouth | 63 ± 10 [47 - 80] |  |
| 50 l min^-1^ closed mouth | 90 ± 6 [72 - 96] | <0.001 |
| 8 vital capacity breaths  50 l min^-1^ closed mouth | 83 ± 9 [66 - 94] |  |
| *Standard nasal cannula* |  |  |
| 50 l min^-1^ open mouth | 70 ± 13 [35 - 96] | 0.018 |
| 8 vital capacity breaths  50 l min^-1^ open mouth | 59 ± 12 [37 - 77] |  |
| 50 l min^-1^ closed mouth | 88 ± 5 [81 - 96] | <0.001 |
| 8 vital capacity breaths  50 l min^-1^ closed mouth | 78 ± 11 [57 - 95] |  |
